# Mental disorders are no predictors to determine the duration of cannabis-based treatment for chronic pain

**DOI:** 10.3389/fpsyt.2022.1033020

**Published:** 2023-01-06

**Authors:** Caroline Rometsch, Stephan Ott, Teresa Festl-Wietek, Anna-Maria Jurjut, Barbara Schlisio, Stephan Zipfel, Andreas Stengel, Anne Herrmann-Werner

**Affiliations:** ^1^University Hospital and Faculty of Medicine, University of Tübingen, Tübingen, Germany; ^2^Department of Experimental and Clinical Medicine, University of Florence, Florence, Italy; ^3^Tübingen Institute for Medical Education, University of Tübingen, Tübingen, Germany; ^4^Department of Anesthesiology and Intensive Care Medicine, Tübingen University Hospital, Tübingen, Baden-Württemberg, Germany; ^5^Department for Psychosomatic Medicine, Charité Center for Internal Medicine and Dermatology, Corporate Member of Freie Universität Berlin, Humboldt-Universität zu Berlin and Berlin Institute of Health, Charité – Universitätsmedizin Berlin, Berlin, Germany

**Keywords:** bio-psycho-social model, cannabis, cannabis-based medicine, chronic pain, depression, mental disorders, pain disorder, treatment

## Abstract

**Background:**

Chronic pain (CP), a complex biopsychosocial disorder with a global prevalence of up to 33%, can be treated by following multidisciplinary approaches that may include cannabis-based medicine (CBM). However, because CBM continues to be a new treatment, questions remain regarding the ideal duration for CBM and its psychosocial determinants, including mental comorbidities.

**Methods:**

In a retrospective cross-sectional study involving 46 patients with CP (ICD-10 code F45.4-), three validated instruments—the German Pain Questionnaire, the Depression Anxiety Stress Scale (DASS), and the Marburg Questionnaire of Habitual WellBeing—were used to identify pain-specific psychosocial determinants and mental disorders. Descriptive analyses, a group differences analysis, and a logistic regression analysis were performed using SPSS.

**Results:**

The patients most frequently reported low back pain as the primary location of their CP, and in attributing the condition to tissue damage, most had largely adopted a somatic orientation in conceptualizing their illness. Most had experienced CP for more than 5 years (*M* = 5.13 years, SD = 1.41) and, as a consequence, faced significant restrictions in their everyday life and exhibited low subjective wellbeing (MFHW median = 4.00, *N* = 43, Q1: 2.00, Q3: 9.00, range: 0–20). Comorbidities among the patients included depression, (DASS-Depression, median: 11.50, Q1: 7.00, Q3: 16.25), anxiety (DASS-Anxiety, median: 4.50, Q1: 2.75, Q3: 8.00), and stress (DASS-Stress, median: 11.00, Q1: 7.00, Q3: 15.00). Between the two cannabis-based treatments with a course lasting either less or more than a year, the duration of treatment showed no between-group differences in terms of sociodemographic factors, pain-specific factors, conceptualizations of the illness, or mental disorders. Psychosocial determinants such as subjective wellbeing and mental comorbidities were not significant predictors of the duration of cannabis-based treatment.

**Conclusion:**

We found no evidence indicating that the benefits of short-term vs. long-term cannabis-based treatment can be predicted by mental comorbidities or psychosocial factors. However, because CBM may be included in approaches to treat CP, questions about the ideal duration of such treatment remain to be answered.

## Introduction

Chronic pain (CP) is a complex but common biopsychosocial disorder that causes severe personal and socioeconomic sequelae for each individual (1). As a consequence, CP is associated with increased 10-year mortality (2) and has been identified as a leading cause of years lived with disability (3). From a socioeconomic perspective, low back pain is the most costly cause of occupational health problems (4) and, for Germany’s healthcare system, has an annual cost of approximately €38 billion due to sick days, on-the-job accidents, and pensions (5). In a systematic review, researchers have estimated the total costs attributable to low back pain to range from $84.1 to $624.8 billion in the United States (6).

The International Association for the Study of Pain defines *pain* as “an unpleasant sensory and emotional experience associated with, or resampling that associated with, actual or potential tissue damage” (7). The fifth edition of the *Diagnostic and Statistical Manual of Mental Disorders*, however, has replaced the term “somatoform disorder” with “somatic symptom and related disorders,” which includes pain with the specifier “with predominant pain” in the category of somatic symptom disorders (8). In Germany, the prevalence rate of CP is 17% (5), whereas studies have revealed an even higher prevalence in the United States, namely, 20% (9). Worldwide, CP of any type occurs with prevalence rates of approximately 33% (10). The etiology of CP, however, remains incompletely understood. Several studies have identified the condition’s risk factors (11) and relevant somatic processes, such as central pain amplification (12, 13).

Because CP’s comorbidity with mental disorders, such as depression (14), and co-occurrence with anxiety disorders are well-documented (15), studies have focused on prevention strategies (16) and treatment options (17) for the condition. Such work suggests that treating CP requires a multidisciplinary pain management program following a biopsychosocial approach (17). Past investigations have especially described CP management that, as part of a multidisciplinary approach (18), includes addressing social determinants (19). Such multimodal pain therapy consists of somatic and psychotherapeutic procedures such as physical and psychological therapies (20), with pharmacological therapy with opioids (21) or antidepressants (22) as an additional therapeutic strategy (23). Although cannabis-based medicine (CBM) has been discussed as being efficient in reducing CP, evidence of its effectiveness remains limited (24), especially due to a risk profile containing adverse reactions (25). As an adjuvant treatment, CBM is often used in combination with opioids (26) and, for that reason, is often a last pharmacological resort for treating CP. Despite recent reviews mostly focused on CBM in comorbid mental disorders (27), evidence remains inconclusive about the most effective time point to use CBM to treat patients with CP and the most adequate treatment duration. Beyond that, little is known about CBM’s efficacy with comorbid mental disorders and other psychosocial determinants.

## Aim

This study aimed to obtain reliable evidence on the treatment with CBM in CP management that requires identifying the determinants, if any, by following a biopsychosocial approach. To that end, in our investigation, we sought to identify a suitable duration for the treatment using CBM, as well as important associations between the duration and both mental disorders and psychosocial determinants among patients with CP.

## Materials and methods

### Study design

In a retrospective cross-sectional investigation designed to identify associations between the duration of cannabis-based treatment and psychosocial determinants as well as mental disorders among patients with CP, we first screened patients’ files for eligibility. Although 130 patients met the criteria to participate, due to missing data from validated psychometric instruments, 83 patients were excluded from our analysis. Thus, we analyzed data from 47 patients, all of whom completed validated psychometric instruments. Patients’ data were pseudonymized by using random participant codes, and the study received ethics approval from the University of Tübingen (No. 578/2021BO2).

### Sample description

Participants were male and female patients with CP being treated with CBM in the outpatient pain clinic within the Anesthesiology Department of the University Hospital of Tübingen. The treatment spanned from April 2017 to August 2019, during which time patients received individually described CBM with diverse applications after consulting with an anesthesiology specialist. All patients were diagnosed with CP according to the ICD-10, which defines *CP* as a condition of pain lasting more than 6 months (28), and there were no restrictions placed upon diagnosis in terms of the localization, type, or characteristics of the pain.

### Data collection

We collected retrospective data from patients in an outpatient pain clinic of the Anesthesiology Department of the University Hospital of Tübingen. Data were extracted from the patient’s records for the period from April 2017 to October 2019.

### Instruments

As is routine, participating patients in the outpatient pain clinic from the Anesthesiology Department of the University Hospital of Tübingen completed validated assessment instruments. The battery consisted of the pain-specific Deutscher Schmerzfragebogen (“German Pain Questionnaire,” DSF) (29), the Depression Anxiety Stress Scale (DASS) (30), and the Marburg Questionnaire of Habitual WellBeing (MFHW) (31).

### German pain questionnaire (DSF)

The DSF (29), widely used in Germany, is an assessment instrument developed by the German Pain Society to assess several determinants of CP on a four-point Likert scale ranging from 0 (*not at all*) to 3 (*exactly*) by asking patients to rate the kind of pain, its location, intensity, their conceptualization of the illness and causal attribution(s), the duration of the pain, and its effects. The kind of pain is assessed by having patients rate 12 adjectives (e.g., “pushing,” “dull,” “sharp,” “terrible,” “hot,” and “throbbing”) with their pain. Pain-associated factors are also assessed, including factors that improve or aggravate the pain. As for the pain’s location, patients are asked to draw the pain on a figure of the body. Meanwhile, pain’s intensity is assessed according to restrictions in everyday life using Von Korff et al.’s Chronic Pain Grade (32), an instrument with five scales used to classify the severity of CP into five grades (32): Grade 0 (i.e., no pain), Grade 1 (i.e., low restrictions), Grade 2 (i.e., high intensity of pain with low restrictions), Grade 3 (i.e., high restrictions with moderate possibilities for limitations), and Grade 4 (i.e., high restrictions and limitations in everyday life) (33). Of those grades, Grades 3 and 4 describe high restrictions in everyday life due to CP (32). Cronbach’s alpha for Von Korff et al.’s Chronic Pain Grade at 0.89 is very good. Next, the duration of pain is assessed by asking patients for a history of the treatment for their pain, including the doctors who treated the pain, the medications that they prescribed, and/or the operations that they performed. Last, the DSF also collects sociodemographic data and other data regarding the patients’ social situation (e.g., employment status, pension status, and degree of disability).

### Depression anxiety stress scale (DASS)

The Depression Anxiety Stress Scale (30) is an assessment instrument with 21 items divided equally across three subscales: depression, anxiety, and stress. Each item is to be rated from 0 (*not at all*) to 3 (*extremely* or *most of the time*), for a maximum total score of 21. A cutoff of 6 is suggested for anxiety and 10 for depression and stress (34). The German version of the DASS can be considered to be a reliable questionnaire, and Cronbach’s alpha for all scales in our sample at 0.81 was very good.

### Marburg questionnaire of habitual wellbeing (MFHW)

The Marburg Questionnaire of Habitual Wellbeing (31) (i.e., FW7), with seven items to be rated on a six-point Likert scale ranging from 1 (no agreement) to 6 (*fully agreement*), is designed to assess positive aspects of mental health. The maximum total score on the MFHW is 35. The instrument’s psychometric quality has been assessed and found to be good (29), and in our sample, Cronbach’s alpha at 0.89 was very good as well.

### Statistical analysis

Sociodemographic data were analyzed using SPSS version 28.0.0 (35), which yielded means and standard deviations. Because the data for group comparison did not follow normal distribution (36, 37), in this study, we report medians and quartiles, and we used non-parametric tests in our analyses. Chi-square tests were conducted for categorical variables and Mann–Whitney *U*-tests for group comparisons with interval-scaled variables. All *p-*values less than 0.05 were considered to indicate statistical significance. A logistic regression analysis was chosen due to the dichotomy of the duration of cannabis-based treatment (i.e., cannabis-based treatment for less than a year vs. cannabis-based treatment for more than a year), which served as the dependent variable.

## Results

### Sociodemographic data

The mean age of the patients in the sample was 52.91 years (SD = 13.86, range: 24–77), and 22 patients (46.8%) were male. Regarding socioeconomic characteristics, 27 patients (57.1%) were not employed, four patients (8.5%) reported being unable to work, and 22 patients (46.8%) reported a pension due to work-related disability. Further information is shown in Table 1.

**TABLE 1:** Sociodemographic data of patients.

	*n*	%
**Age at beginning of cannabinoid therapy**	**42**	
21–25 years	1	2.4
26–35 years	3	7.1
36–45 years	11	26.2
46–60 years	12	28.6
> 60 years	15	35.7
**Sex**	**47**	
Female	25	53.2
**Nationality**	**47**	
German	45	95.7
Greek	1	2.1
Italian	1	2.1
**Current living situation**	**47**	
Alone	11	23.4
With spouse or partner	31	66.0
With children and their spouse or partner	14	29.8
**Education**	**47**	
No degree	1	2.1
Secondary modern school until 9th grade	17	36.2
Secondary modern school until 10th grade	16	34.0
High school until 12th grade	6	12.8
High school until 13th grade	7	14.9
**Pension or disability**	**47**	
Receiving a pension	22	46.8
Planning to apply for pension	6	12.8
Applied for pension without decision	6	12.8
Denial of pension application	1	2.1
Recognized degree of disability	30	63.8
< 50% disability	17	43.6
> 50% disability	13	56.4

### Duration of cannabis-based treatment

The average duration of cannabis-based treatment was more than a year (*M* = 456.95 days; SD = 227.91); 22 patients (56.4%) received the treatment for less than a year and 17 patients (43.6%) for more than a year. We found no significant differences in the duration of cannabis-based treatment and the sociodemographic variables of gender [χ^2^ (1, *n* = 34) = 0.642, *p* = 0.725, Cramér’s *V* = 0.134], level of education [χ^2^ (1, *n* = 34) = 0.269, *p* = 0.966, Cramér’s *V* = 0.089], and pension [χ^2^ (1, *n* = 30) = 0.344, *p* = 0.558, Cramér’s V = 0.107].

### Duration and intensity of pain duration

**FIGURE 1:**
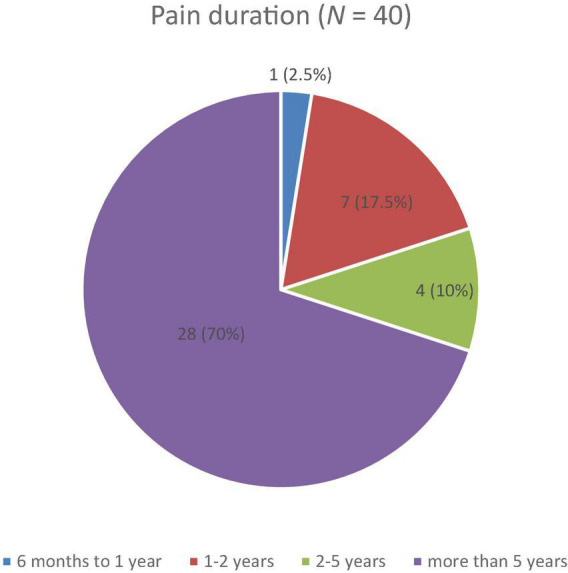
Reported duration of pain among patients with chronic pain on a four-point Likert scale with durations of 6 months to 1 year, 1–2 years, 2–5 years, and more than 5 years.

Descriptive analyses revealed that most patients in the sample (*n* = 28, 70.0%) reported suffering from CP for more than 5 years (*M* = 5.13, SD = 1.41), as shown in Figure 1, the duration of symptoms that far exceeds the threshold of 6 months of pain set by the ICD-10.

**FIGURE 2:**
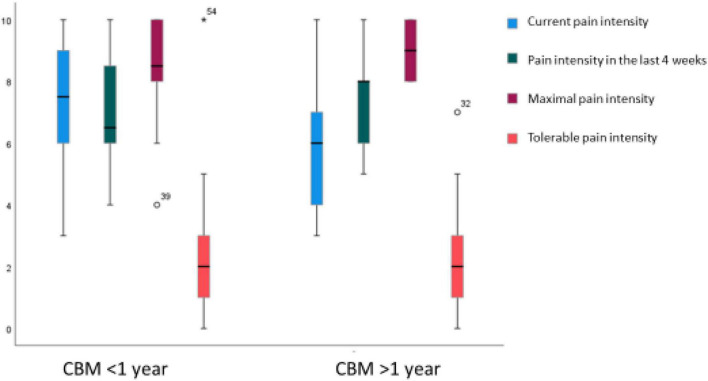
Distribution of pain intensity at present, in the last 4 weeks, maximal pain intensity, and tolerable pain intensity for groups who received treatment with cannabis-based medicine (CBM) for less vs. more than a year.

Approximately a third of the sample reported suffering from persisting undulating pain. Another third described suffering from CP marked by sporadic attacks of pain. The patients’ reported intensity of pain is shown in Figure 2. No significant group-based differences arose between cannabis-based treatment lasting for more than a year and less than a year in terms of the intensity and duration of pain.

### Location of pain

The sample reported suffering predominantly from low back pain (*N* = 33, 70.2%), and some also had complaints of joint pain (*n* = 23, 48.9%), pain in the extremities (*n* = 23, 48.9%), head or face pain (*n* = 13, 27.7%), and abdominal pain (*n* = 12, 25.5%). Data from the DSF were analyzed with the duration of the cannabis-based treatment, the results of which are shown in Table 2.

**TABLE 2:** Locations of pain targeted by cannabis-based treatment lasting for more and less than a year.

	CBM less than 1 year (*n* = 22)	CBM more than 1 year (*n* = 17)
Low back pain	17(77.3%)	11(64.7%)
Joint pain	14(63.6%)	8(47.1%)
Pain in the extremities	14(63.6%)	6(35.3%)
Head or face pain	7(31.8%)	5(29.4%)
Abdominal pain	6(27.3%)	4(23.5%)

We found no significant difference in cannabis-based treatment for less or more than a year with the location of pain: low back pain [χ^2^ (1, *n* = 34) = 0.019, *p* = 0.891, Cramér’s *V* = 0.023]; joint pain [χ^2^ (1, *n* = 34) = 2.847, *p* = 0.092, Cramér’s *V* = 0.289]; head or face pain [χ^2^ (1, *n* = 34) = 0.200, *p* = 0.655, Cramér’s *V* = 0.077]; and abdominal pain [χ^2^ (1, *n* = 34) = 2.949, *p* = 0.114, Cramér’s *V* = 0.271].

### Treatment history

Of all patients, 34 (87.2%) had undergone an operation, whether an arthroscopy of the shoulder, knee, herniated disk, or endoprosthesis. Most often, treatment for the patient’s CP involved taking medication, including non-steroidal antirheumatics (e.g., metamizole and ibuprofen), opioids (e.g., tilidine, tramadol, and morphine), and/or anticonvulsants (e.g., pregabalin). Patients had been treated in the past with a median of 4.00, different medications each (Q1: 3.00, Q3: 6.00, range: 1–13).

### CP’s impact on quality of life

**FIGURE 3:**
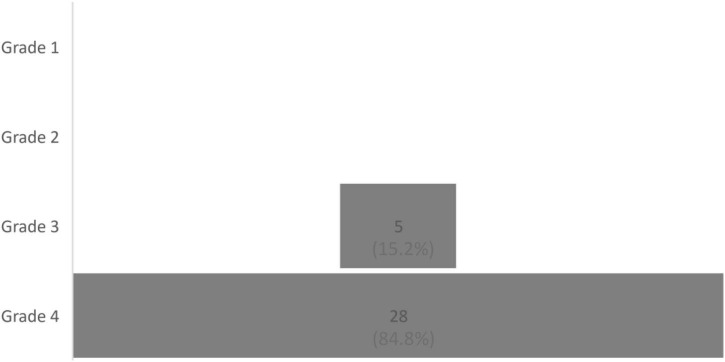
Distribution of patients according to the four grades of Von Korff et al.’s Chronic Pain Grade, *N* = 33.

Due to CP, patients reported having been restricted in their daily activities in the past 3 months for approximately 80.00 days (*n* = 37, Q1: 25.50; Q3: 90.00, range: 0–92), with a median of 8 (*N* = 44, Q1: 5.00; Q3: 9.00, range: 0–10) on a visual analog scale ranging from 0 (*no restrictions*) to 10 (*extremely high restrictions*). The patients also reported being restricted in their work functioning, with a median of 8.00 (*N* = 44, Q1: 7.00; Q3: 10.00, range: 0–10). However, we again found no between-group difference for the duration of the cannabis-based treatment in relation to daily restrictions (restrictions in daily activity: *U* = 114.00, *z* = −0.82, *p* = 0.441; restrictions in work activity: *U* = 91.00, *z* = −1.28, *p* = 0.223; and restrictions in the number of days without experiencing debilitating pain, *U* = 79.00, *z* = −0.55, *p* = 0.614). The distribution for disability using Von Korff’s index is shown in Figures 1–3.

### Conceptualizations of illness with CP

**FIGURE 4:**
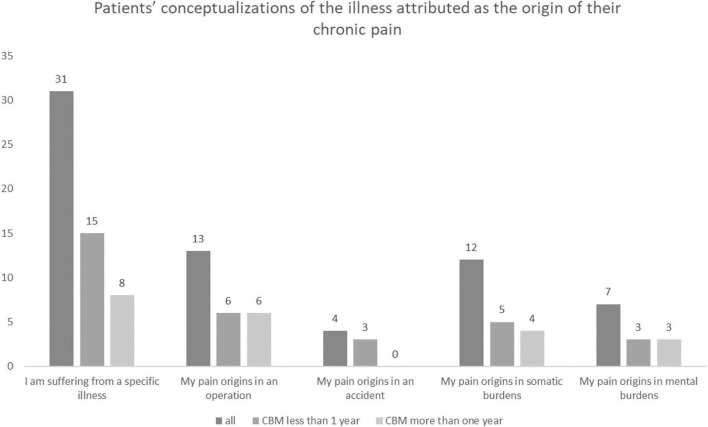
Patients’ conceptualizations of the illness attributed as the origin of their CP, both overall and according to the duration of treatment with cannabis-based medicine (CBM).

Most of the patients (*n* = 44, 88.6%) could identify a reason for their pain, the details of which are illustrated in Figure 4. We did not find any between-group differences in the conceptualizations of the illness with the duration of the cannabis-based treatment.

### Subjective wellbeing

The analysis of the MFHW revealed a median of 4.00 (*n* = 43, Q1: 2.00, Q3: 9.00, range: 0–20). Patients with CP and cannabis-based treatment lasting less than a year had a median of 6.00 (*n* = 20, Q1: 0.50, Q3: 17.00, range: 0–20), whereas those who had received the treatment for more than a year had a median of 4.00 (*n* = 13, Q1: 1.50, Q3: 8.00, range: 0–12). There was no significant between-group difference between the duration of cannabis-based treatment and MFHW score (*U* = 107.50, *z* = −0.83, *p* = 0.413).

### Mental disorders

**FIGURE 5:**
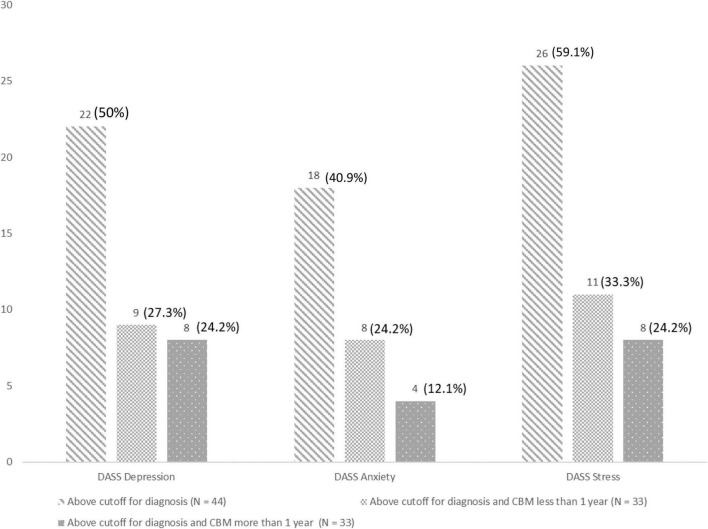
Results of the depression anxiety atress scale (DASS) in terms of the depression, anxiety, and stress subscales for the sample overall and in relation to the duration of cannabis-based medicine (CBM) treatment (i.e., less or more than a year).

Analyses of the DASS data revealed a median score on the depression subscale of 11.50 (*N* = 42, Q1: 7.00, Q3: 16.25, range: 0–20), on the anxiety subscale of 4.50 (*N* = 42, Q1: 2.75, Q3: 8.00, range: 0–18), and the stress subscale of 11.00 (*N* = 42, Q1: 7.00, Q3: 15.00, range: 1–19). The distribution for patients who were above the cutoff to be diagnosed with the mental disorder is shown in Figure 5. We found no significant between-group differences between the duration of cannabis-based treatment and the presence of mental disorders (depression: *U* = 104.00, *z* = −0.83, *p* = 0.427; anxiety: *U* = 114.50, *z* = −0.215, *p* = 0.883; stress: *U* = 136.00, *z* = −0.02, *p* = 0.986).

### Regression analysis

To determine the predictive value of mental health on the dependent variable (i.e., duration of cannabis-based treatment), we used a binary logistic regression model. Both the MFHW and the DASS, including all three of its subscales (i.e., depression, anxiety, and stress), remained non-significant predictors, as shown in Table 3.

**TABLE 3:** Results of the binary linear regression model to assess the predictive value of the sepression anxiety stress scale (DASS) and the marburg questionnaire of habitual wellbeing (MFHW) for the dependent variable of cannabis-based treatment lasting less or more than a year.

	Odds ratio	95% confidence interval	*p*
Constant	1.774		0.550
MFHW	0.891	(0.76, 1.04)	0.891
DASS depression	0.941	(0.77, 1.15)	0.550
DASS anxiety	1.049	(0.82, 1.34)	0.706
DASS stress	0.997	(0.79, 1.26)	0.979

## Discussion

This article offers valuable insights into CBM as a commonly used treatment for patients suffering from CP. In particular, it answers essential questions regarding the impact of the duration of cannabis-based treatment lasting either more or less than a year, as well as questions regarding associations between the duration of such treatment and both mental illnesses and psychosocial determinants. In our study, we aimed to identify for the first time the effectiveness of short-term and long-term (i.e., less and more than a year, respectively) cannabis-based treatment and compared those durations with mental disorders and determinants of the psychosocial environment to ultimately generate theoretical and practical implications for the treatment of CP.

Assessments of mental disorders and determinants as psychosomatic and psychosocial variables that may influence cannabis-based treatment remain rare (38), despite considerable interest in the effectiveness of medical cannabis and mental health (39). Symptoms of pain constitute the dominant somatic burden for the medical use of cannabis (40). To gain insight into the necessary duration for CBM, in our study, we analyzed a sample of patients with CP. Our investigation focused on the duration of cannabis-based treatment, with a close look at the effect of receiving CBM for CP for less or more than a year. To the best of our knowledge, few studies have investigated the efficacy of short-term vs. long-term cannabis-based treatment and mental disorders. However, large systematic reviews have revealed considerable heterogeneity regarding the duration of treatments in research, ranging from 1 to 26 weeks, and provided evidence depending on which CBM is used (41, 42). Our sample was relatively heterogeneous in terms of the CBM reported, although our investigation did not reveal evidence between short-term and long-term cannabis-based treatment and our sociodemographic data points. We also did not find any evidence of a relationship between the duration of cannabis-based treatment and gender or level of education. In the literature, unemployment and lack of work autonomy are shown to be important predictors of CP, along with factors such as job satisfaction, additionally, difficult job conditions are associated with disability due to CP (43). Regarding pain management with CBM, we also did not find that the duration of treatment with CBM differs according to employment or pension status, which is somehow surprising given that cannabis-based treatment for CP generally leads to improved quality of life (44, 45), not only for patients with CP but also for patients with other chronic conditions (46). Presumably, long-term cannabis-based treatment should also influence one’s ability to work. However, our analyses revealed that the majority of the sample reported suffering from a Von Korff index of 3 or 4, and in both groups receiving cannabis-based treatment, patients were significantly restricted in their ability to work. Focusing on other daily restrictions, we could not identify any difference between the groups. Receiving CBM for more than a year did not improve patients’ abilities in everyday life relative to the abilities of patients who received CBM for less than a year.

Our analyses also revealed that the majority of patients with CP had suffered from low back pain with high intensity for more than 5 years. Large systematic reviews report back pain as being the most frequent complaint of patients with CP (47), one with increased prevalence beginning in the third decade of life (48). Regarding the duration of cannabis-based treatment, however, our analyses of the location of pain did not show any significant between-group differences. Moreover, and probably of more urgent relevance for both patients and practitioners, we found that the treatment durations of both less than and more than a year did not change the perception of the intensity of pain. The literature shows, on the one hand, that cannabis-based treatment among patients with CP reduces the intensity and severity of pain (49, 50). Similar results emerged from a cohort study whose participants were regularly observed while taking a 1-year course (51), although those results remain controversial and criticized due to the limited amount of evidence (52). On the other hand, in the context of long-term CBM therapy, increases in the intensity of pain have also been reported (53), with results confirmed with reference to evidence concerning diverse somatic pathological conditions (54). Our results indicate that receiving CBM for either less or more than a year is not a reliable marker for identifying changes in the perception of pain. Our analyses revealed that patients with CP used medications intensively in the past, sometimes with more than four different analgesics, and half of our sample population had an operation. In the vast majority of cases, CP is treated by medication (55). Opioids are the most used analgesics, with prevalence rates of up to 81% (56), despite only slight reductions in pain and improvements in physical functioning, as revealed by large meta-analyses (21). Furthermore, the increased risk of adverse reactions to opioids has to be taken into account (21).

For an in-depth understanding of the perception and processing of pain, conceptualizations of illness may offer insights into patients’ experiences. Thus, our analysis of conceptualizations of illness (57) is important, as such conceptualizations influence several treatment outcomes, particularly because they impact help-seeking strategies (58). Patients in our sample reported somatic-oriented explanations, that is, beliefs that their symptoms originate in a somatic dysfunction or tissue damage. Patients also attributed their pain to specific illnesses, operations, accidents, or other somatic burdens instead of mental conditions, which may lead to further excesses in medical care (59). The somatic orientation as a style of attribution and conceptualization of illness can be explained by the high number of somatic comorbidities in our sample. Therefore, we assumed that patients with CP more often develop somatic conceptualizations and therefore focus on somatic-oriented explanations for their pain. However, we did not find any change in the patients’ conceptualizations of illness with the duration of cannabis-based treatment. CBM does not seem to influence the style of attribution, at least not in a course lasting a maximum of 2 years.

In general, beliefs about pain, next to depressive mood, seem to be highly predictive of disability (60). In addition, direct associations between quality of life and stress-related symptoms have been found in patients with CP (61). Even though most of our sample did not report being diagnosed with a psychiatric condition prior to participating in our study, our investigation revealed evidence of severe depression and anxiety among the patients sampled. Those results help to answer extensively studied research questions concerning the comorbidities of CP. Considerable evidence of depression (14) and anxiety disorders (15) as comorbid mental disorders has been found in patients suffering from CP. Detailed mechanisms between anxiety and CP have also been studied intensively, namely, with anxiety sensitivity and fear of pain as relevant determinants of pain-related avoidance behavior (62). Our results also emphasize the relevance of adopting a clinical focus when assessing determinants of pain to identify possible anxiety disorders. Beyond that, chronic stress may be a linking factor between CP and depression (63), as described in animal models (64), which would explain our findings with the DASS.

Regarding the duration of cannabis-based treatment, patients receiving CBM for more than a year did not differ significantly from patients with short-term cannabis-based treatment. To the best of our knowledge, no literature answers the question of which duration of treatment with CBM may improve mental comorbidities. From a clinical perspective, our findings underscore the importance of taking a multidisciplinary approach to managing CP (18). In pharmacological treatments, comorbid CP and mental disorders often complicate decisions about the class of substance to be administered, even though serotonin–norepinephrine reuptake inhibitors, tricyclic antidepressants, and anticonvulsants have shown efficacy (65), as has CBM (66), in the case of the comorbidity of CP with a mental disorder. With our findings, we have shown that there is no difference in comorbidities in cannabis-based treatment lasting more or less than a year. On top of that, mental disorders and the subjective wellbeing of CP were not significant predictors of the duration of the cannabis-based treatment.

Finally, our results should be viewed in the overall context of the position paper of European Pain, an expert group that empowers and informs specialists. The group has advocated cannabis-based treatment as an adjunctive medication as part of multidisciplinary treatment, particularly when guideline-recommended therapies are not successful (67). Because gaps in the literature addressing CBM in patients with comorbid mental disorders remain, the use of CBM, including its duration, should be adapted to each individual and each individual monitored, and goals regarding the duration and termination of the treatment should be predefined (67).

## Limitations

First, our study design is a retrospective analysis. However, by using that design, we were able to investigate a sample of patients with CP with a cannabis-based treatment that can be regarded as long-term, research on which remains sorely limited. At the same time, our sample size was rather small, we could not analyze causality with our study design, and we used self-report measurements, even though they are considered to be validated and are implemented regularly as assessment instruments. In light of those limitations, a longitudinal investigation would be clinically interesting for consolidating the outcomes of CBM and analyzing psychosomatic development. An analysis with another sample in Tübingen, also with treatment as usual (i.e., without CBM), would be especially valuable.

## Conclusion

Cannabis-based medicine is a treatment option in the framework of multimodal treatment approaches for CP. Pain-specific, psychosocial factors, and mental comorbidities are indisputable factors in the success of CBM therapy. At the same time, our results do not implicate any benefit of those factors in the case of cannabis-based treatment lasting less than a year compared with treatment lasting more than a year. Neither mental comorbidities nor the patients’ subjective wellbeing predictors were able to differentiate treatment duration. Research into possible relationships between those determinants and cannabis-based treatment duration, therefore, remains necessary.

## Data availability statement

The original contributions presented in this study are included in the article/supplementary material, further inquiries can be directed to the corresponding author.

## Ethics statement

The studies involving human participants were reviewed and approved by University of Tübingen (number: 578/2021BO2). The patients/participants provided their written informed consent to participate in this study.

## Author contributions

AH-W, and TF-W designed the study with support of BS and AS. CR analyzed the data and wrote the manuscript with support of TF-W, SO, A-MJ, and AH-W. SZ, AS, and BS supported with scientific suggestions on the manuscript. All authors contributed to the article and approved the submitted version.
